# Addition of Multimodal Therapy to Standard Management of Steady State Sickle Cell Disease

**DOI:** 10.1155/2013/236374

**Published:** 2013-12-09

**Authors:** Iheanyi Okpala, Osita Ezenwosu, Anthony Ikefuna, Augustine Duru, Barth Chukwu, Anazoeze Madu, Theresa Nwagha, Sunday Ocheni, Obike Ibegbulam, Ifeoma Emodi, Uche Anike, Charles Nonyelu, Chukwudi Anigbo, Kingsley Agu, Ifeoma Ajuba, Awele Chukwura, Ogechukwu Ugwu, Uche Ololo

**Affiliations:** ^1^Department of Haematology, University of Nigeria Teaching Hospital, Enugu 46000, Nigeria; ^2^Department of Haematology, College of Medicine, University of Nigeria, Enugu Campus, Nigeria; ^3^Department of Paediatrics, University of Nigeria Teaching Hospital, Enugu 46000, Nigeria

## Abstract

Most people on folic acid to boost erythropoiesis and prophylactic antimicrobials, the standard management of steady state sickle cell disease (SCD), have unacceptable numbers of crises. The objective of this study was to evaluate the effects of adding multimodal therapy with potassium thiocyanate and omega-3 fatty acids to the standard management of steady state SCD. Pre- and post-treatment numbers of crises and other disease indices were compared in 16 HbSS individuals on folic acid and paludrine after 12 months of adding eicosapentaenoic acid 15 mg/kg/day, docosahexaenoic acid 10 mg/kg/day, and potassium thiocyanate 1-2 mL/day, each milliliter of which contained 250 mg of thiocyanate and 100 micrograms of iodine to prevent hypothyroidism: a possible side-effect due to competitive inhibition of the transport of iodide into the thyroid gland by thiocyanate. Median number of crises reduced from 3/yr to 1/yr (*P* < 0.0001). There was no evidence of impaired thyroid function. Plasma level of tri-iodothyronine improved (*P* < 0.0001). Steady state full blood count and bilirubin level did not change significantly. The findings suggest that addition of potassium thiocyanate and eicosapentaenoic and docosahexaenoic acids to standard management of steady state SCD reduces the number of crises. This observation needs to be evaluated in larger studies.

## 1. Introduction

The inherited blood condition sickle cell disease (SCD) affects 20–25 million people worldwide and constitutes a major health problem on a global scale. This multiorgan disease is characterised by crescent-shaped (sickle) red blood cells, premature destruction of erythrocytes (haemolysis) resulting in anaemia, susceptibility to infections, and recurrent obstruction of blood vessels which causes tissue ischaemia or infarction—the pathological process underlying the episodes of generalised (ischaemic) pain called vasoocclusive crisis. The period of relative good health between crises is referred to as “steady state.” Sickle cell disease in steady state is currently managed with folic acid to boost production of red blood cells, antimicrobial drugs to prevent infections, and, in severely affected individuals who constitute less than 5% of all patients, either haemopoietic stem cell transplantation or the cytotoxic drug hydroxycarbamide (hydroxyurea) which increases the proportion of foetal haemoglobin inside erythrocytes and so inhibits sickling. The majority of people with SCD who are neither on hydroxyurea nor have had haemopoietic stem cell transplant (over 95%) still have considerable numbers of crises despite taking folic acid and prophylactic antimicrobials.

A therapeutic intervention that disrupts at least one of the fundamental mechanisms of sickle cell disease—blood vessel occlusion, haemolysis, and impaired immunity—has the potential to ameliorate the condition. Although the mechanisms and pathogenesis of SCD are fairly well understood, there has been no comparable progress in developing a generally acceptable and affordable treatment that has clinical efficacy. To date, specific treatment of SCD in steady state has been with agents that disrupt one of the fundamental mechanisms of the disorder (single modality therapy), such as the administration of an antisickling agent. The clinical benefit of single modality therapy which inhibits only one of the three fundamental mechanisms of SCD is limited by the fact that the other 2 mechanisms continue to cause organ damage and dysfunction. Therefore, there is need to explore new approaches to specific treatment of steady state SCD, in order to advance beyond the level of efficacy attainable by the current standard management using folic acid and prophylactic antimicrobials. Multimodal therapy is a novel approach that combines various agents each of which inhibits a different mechanism of SCD. An example of multimodal therapy is the combined administration of omega-3 fatty acids and potassium thiocyanate. The omega-3 fatty acids (eicosapentaenoic acid, EPA, and docosahexaenoic acid, DHA) are important structural and functional constituents of the red blood cell membrane which have been shown to inhibit haemolysis and vasoocclusion, thereby reducing the number of vasoocclusive crises in SCD [1–3]. Potassium thiocyanate is a constituent of foods such as yam (*Dioscorea*) and inhibits sickling of red blood cells [4]. The rationale for multimodal therapy with thiocyanate, EPA, and DHA is that, by inhibiting blood vessel occlusion, erythrocyte sickling, and haemolysis, these naturally occurring substances might have additive or synergistic effects. The multimodal approach to the specific treatment of steady state SCD is analogous to combination chemotherapy of cancers with cytotoxic drugs each of which inhibits cell proliferation by a different mechanism.

We report the findings from a pilot evaluation of the effects of adding potassium thiocyanate, EPA, and DHA to standard management of steady state SCD with folic acid and paludrine. By inhibiting more than one mechanism of SCD these omega-3 fatty acids and potassium thiocyanate have the potential to achieve greater clinical efficacy than folic acid and paludrine. DHA and EPA have important clinical and socioeconomic advantages over hydroxycarbamide in that they are neither cytotoxic nor potentially teratogenic and are more widely available and affordable in low-medium resource countries where the majority of SCD patients live. As naturally occurring food substances present in fish oil taken in many parts of the world, EPA and DHA are more culturally acceptable than hydroxycarbamide in various communities because of their proven long-term safety.

## 2. Methods

This investigation was done according to the ethical standards laid down in the 2008 (Seoul) revision of the 1964 Declaration of Helsinki. The study was designed to compare various indices of SCD before and after the addition of multimodal therapy with EPA, DHA, and potassium thiocyanate. Since wide interperson variation in clinical and laboratory indices is a prominent feature of SCD [5, 6], the study was designed to reduce the confounding effect of individual variability, and each participant was his/her own control. Following Institutional Review Board approval and informed consent, 16 persons who have homozygous (HbSS) sickle cell disease and met the eligibility criteria were randomly enlisted for the study. The participants were 7 adults and 9 children (11 males and 5 females) of age 3 yrs–33 yrs. The inclusion criterion was occurrence of 3 or more episodes of sickle cell crisis that required hospital admission (in-patient management) in the previous 12 months. The exclusion criteria were conditions known to affect the indices of SCD, such as a programme of blood transfusion therapy, current or prior treatment with omega-3 fatty acids or hydroxycarbamide (hydroxyurea), pregnancy in the previous 3 months, and coexistence of any other chronic illness (e.g., asthma or diabetes mellitus) in the HbSS person.

Each participant continued on folic acid 3–5 mg and paludrine 100–200 mg all through the period of study. In addition, they had EPA 15 mg/kg/day and DHA 10 mg/kg/day. These omega-3 fatty acids were taken by mouth as capsules of fish oil extract each gram of which contains EPA 180 mg, DHA 120 mg, and 2 IU of the antioxidant alpha tocopherol (vitamin E) to inhibit oxidation of EPA and DHA. A liquid formulation of potassium thiocyanate was taken orally at doses that varied according to age. For participants of age >18 yrs the dose of potassium thiocyanate was 2 mL/dy, 9–18 yrs (1.5 mL/dy), 3–8 yrs (1 mL/dy). Each millilitre (mL) of the potassium thiocyanate formulation contains 250 mg of thiocyanate and 100 micrograms of iodine to prevent hypothyroidism: a possible side-effect due to competitive inhibition of the transport of iodide into the thyroid gland by thiocyanate [7]. To assess compliance with taking DHA, EPA, and thiocyanate, study participants were requested to bring the containers (used and still in use) with them on the clinical review visits scheduled at 2-week intervals for paediatric participants during the entire period of study and, for adults, initially at intervals of 2 weeks, increasing to 3 months as the study progressed. On each visit the number of omega-3 fatty acid capsules was counted and the quantity of potassium thiocyanate noted to ensure they were appropriate for the time of review. At the beginning of the study (month 0), on each visit for review, and after 12 months of taking EPA, DHA, and potassium thiocyanate, each participant had clinical evaluation including medical history and review of the hospital records to ascertain the number of sickle cell crises. Pre- and post-treatment full blood count and bilirubin level were measured with an automated blood cell counter and a blood chemistry analyzer, respectively. Apart from the visits scheduled for review, study participants were advised to attend the hospital whenever they developed acute illness (such as sickle cell crisis) or suspected adverse events, so they could be evaluated and given appropriate medical treatment. To assess the effect of potassium thiocyanate on thyroid function, steady state levels of thyroid stimulating hormone (TSH) and tri-iodothyronine (T3) were measured at months 0 and 12 of the study. Enzyme-Linked Immunosorbent Assay (ELISA) kits purchased from Diagnostic Automation Inc., Calabasas, USA, were used to measure plasma concentrations of TSH (Lot no. 112022302) and T3 (Lot no. 111110202) according to the manufacturer's instructions. The Statistical Package GRAPHPAD PRISM, version 8.0 (GraphPad, La Jolla, CA, USA) was used to analyse data generated from the study. For robust interrogation of the data, the nonparametric Mann-Whitney *U* test of significance (which does not assume normal distribution data) was applied. We compared the number of sickle cell crises that occurred within the one year before and after starting multimodal therapy and also months 0 and 12 values of steady state plasma bilirubin concentration, full blood count, TSH, and T3 levels.

## 3. Results

To facilitate direct assessment of the effects of adding the combination of thiocyanate, EPA, and DHA to the current standard management of steady state sickle cell disease, raw data for individual study participants are presented in Tables 1, 2, and 3. Fifteen of the sixteen (15/16) participants (94%) had a reduction in the number of sickle cell crises while on multimodal therapy; see Table 1. This effect was highly significant on statistical analysis (*P* < 0.0001). Steady state haemoglobin level and leukocyte and platelet counts did not change significantly before and after treatment. Pre- and post-treatment concentrations of unconjugated bilirubin in plasma were measured on 12 participants (Table 2) and showed no significant difference. Although plasma concentrations of thyroid stimulating hormone before and after treatment were comparable in the 13 individuals who had assessment of thyroid function (Table 3), there was significant improvement of tri-iodothyronine levels during the period of multimodal therapy (*P* < 0.0001).

## 4. Discussion

The results suggest that addition of multimodal therapy with potassium thiocyanate, EPA, and DHA to folic acid and paludrine during steady state reduces the number of sickle cell crises. There is need to further evaluate the effects of this combination in larger, multicentre studies. The combination of thiocyanate and omega-3 fatty acids includes agents with antisickling, antiadhesion, and cell membrane-stabilizing effects. Therefore, this combination has the potential to inhibit three mechanisms of sickle cell disease—erythrocyte sickling, blood vessel occlusion, and haemolysis. These effects might synergize with each other to enhance the overall clinical benefit of the combination.

Blood vessel occlusion—the major mechanism of organ damage in SCD—results from adherence of blood cells to each other and the wall of the blood vessel, a process mediated by intercellular adhesion molecules. Amongst the molecules that mediate intercellular adhesion and facilitate vasoocclusion in SCD, p-selectin occupies a strategic position because it is the one molecule involved in the adhesion of all three types of blood cells (erythrocytes, leukocytes, and platelets) not only to the vascular endothelium, but also to each other [8, 9]. Administration of EPA and DHA reduces the expression of p-selectin on platelets and is beneficial in SCD [1–3]. In addition, omega-3 fatty acids reduce expression of intercellular adhesion molecule-1 (ICAM-1), leukocyte adhesion to vascular endothelium, and production of four biologically active molecules involved in the pathophysiology of tissue damage in SCD: interleukins (IL)-1*β*, -6, and -8 and tumour necrosis factor-alpha [10]. Furthermore, it has been observed that the greater the quantity of EPA and DHA in the blood, the lower the risk of developing complications of SCD and the lesser the degree of anaemia [11, 12]. This is because EPA and DHA get incorporated in the plasma membrane of erythrocytes and increase the resistance of red blood cells to lysis in saline [13]. Therefore, it is plausible that these omega-3 fatty acids also increase erythrocyte membrane integrity in vivo and, thereby, inhibit haemolysis—the dominant mechanism of anaemia in SCD. Thiocyanate inhibits formation of (the abnormally shaped) sickle cells that contribute to obstruction of blood vessels. From the above, concurrent intake of thiocyanate, EPA, and DHA has the potential to confer greater clinical benefit in SCD than each agent taken singly.

The reduction of number of crises observed in 15 of the 16 participants was not associated with improved haemoglobin level in all individuals (Table 1). This observation is most noticeable in the participants with study identity numbers 3, 13, and 14 and is hardly surprising because the fundamental mechanism of crisis in SCD (vasoocclusion) is different from the dominant cause of anaemia (haemolysis).

Although the clinical severity of SCD increases with steady state leukocyte count [14, 15], the response to multimodal therapy in this study was not dependent on whether or not there was a reduction in the white blood cells. For example, participants numbers 4, 11, 13, and 15 responded clinically with reduced numbers of crises but actually had increased steady state leukocyte counts after treatment. Another biological variable that modulates the severity of SCD is the proportion of foetal haemoglobin (HbF) in the individual [16, 17]. However, HbF levels were not measured as part of this study and it was not possible to ascertain whether response to multimodal therapy depends on the proportion of foetal haemoglobin in the blood. The questions whether response to multimodal therapy is affected by HbF level or degree of reduction in leukocyte count could be addressed in future, preferably larger and multicenter studies evaluating the efficacy of multimodal therapy in sickle cell disease.

From medical, social, and economic perspectives, EPA and DHA have important advantages relative to hydroxycarbamide (hydroxyurea) or a programme of blood transfusion therapy. These omega-3 fatty acids are not cytotoxic, unlike hydroxycarbamide. So there are no concerns about myelosuppression, increased risk of infection, or possible teratogenic effects associated with their intake. Being natural constituents of fish oil, EPA and DHA are more culturally acceptable because they have been safely consumed as food in various parts of the world over the millennia. They are also more affordable than hydroxyurea or a programme of blood transfusion therapy in nonaffluent countries where the overwhelming majority of people affected by sickle cell disease live. Unlike a blood transfusion programme, administration of EPA and DHA carries no risk of development of multiple antibodies to red cell antigens, iron overload, or infection by blood-borne pathogens, known and unknown.

There was no clinical or laboratory evidence from this study that administration of the specific formulation of potassium thiocyanate used as a component of this multimodal therapy is associated with impairment of thyroid function. The pre- and post-treatment plasma concentrations of thyroid stimulating hormone did not change significantly and T3 levels actually improved, plausibly because of iodine supplements in the formulation of thiocyanate taken by the participants. This is important because, as a competitive inhibitor of iodide entry into the thyroid gland, thiocyanate has the potential to reduce biosynthesis of the thyroid hormones (such as tri-iodothyronine or T3) that contain iodine. Therefore, it is noteworthy that no clinical or laboratory evidence of hypothyroidism was found in the participants whose thyroid function was assessed.

There is sound scientific basis for folic acid supplementation and prophylactic antimicrobials during steady state SCD and every reason to continue this practice. Addition of the thiocyanate/EPA/DHA combination (multimodal therapy) is a new avenue that needs further exploration because there is an unmet need to reduce the number of sickle cell crises beyond what is achievable by the current standard management with folic acid and prophylactic antimicrobials.

## Figures and Tables

**Table 1:** Number of sickle cell crises and steady state full blood count in HbSS patients before and 12 months after addition of potassium thiocyanate and omega-3 fatty acids to standard management with folic acid and paludrine.

Study identity number	Age (yrs) and gender	Crisis/year		Hb (g/dL)		WBC × 10^9^/L		Platelets × 10^9^/L	
		Pre-Rx	Post-Rx	Pre-Rx	Post-Rx	Pre-Rx	Post-Rx	Pre-Rx	Post-Rx
1	23 F	6	3	9.3	9.8	14.5	7.2	392	210
2	33 M	15	4	8.3	9.8	11.8	8	315	285
3	19 M	4	2	10	9.1	10	9	100	209
4	26 F	5	2	7.6	8.8	8	9.1	226	240
5	11 M	7	2	6.6	6	9.2	5.6	233	430
6	8 M	3	1	8.9	10	11.6	4.9	197	185
7	14 M	5	1	7.5	8.6	23.8	9.6	495	270
8	9 M	3	1	7.7	7	15	14	373	350
9	5 F	6	1	8	6.7	19.4	11.5	170	195
10	6 M	3	0	8.6	6.5	19.1	6.1	84	130
11	16 M	3	1	8.3	7.6	11.2	20.8	470	358
12	16 M	3	5	7	7	6.4	5.9	240	325
13	18 F	3	0	10	8.1	5.9	10.6	253	263
14	17 M	3	1	7	4.9	17.3	10.3	160	Not done
15	18 F	3	2	6.3	7.4	6.2	10.9	505	350
16	3 M	3	0	7.5	8.3	23.5	17.5	303	388

Median		3	1	7.8	7.8	11.7	9.3	253	270
*P value		<0.0001		0.7		0.08		0.9	

F: female; M: male; Pre-Rx: pretreatment; Post-Rx: posttreatment; *Mann-Whitney *U* test, 2-tailed; pretreatment compared with posttreatment data.

**Table 2:** Steady state plasma levels of unconjugated bilirubin in HbSS individuals before and 12 months after addition of omega-3 fatty acids and potassium thiocyanate to standard management with folic acid and paludrine.

Study identity number	Plasma unconjugated bilirubin level (umol/L)	
	Pretreatment	Posttreatment
1	4.3	4.1
2	7	4.4
3	25.6	11
4	4.2	4.5
7	4	7.7
8	23	9.8
9	4	16.5
10	3	22
11	10	27
12	14	20
13	1	19.3
14	14	21

Median (*n* = 12)	5.65	13.75
*P* value (2-tailed, Mann-Whitney *U* test pre- versus posttreatment data)	0.11 (no significant difference)	

**Table 3:** Steady state levels of plasma thyroid stimulating hormone (TSH) and tri-iodothyronine (T3) in SCD patients before and 12 months after adding omega-3 fatty acids and potassium thiocyanate to standard management with paludrine and folic acid.

Study no.	TSH *μ*IU/mL		Free T3 ng/mL	
	Pretreatment	Posttreatment	Pretreatment	Posttreatment
1	3.65	2.98	0.67	1.4
3	2.25	1.28	0.69	1.14
4	2.31	1.94	0.68	1.2
7	2.84	2.56	0.66	1.37
8	2.23	1.98	0.75	1.46
9	7.05	5.3	0.6	0.99
10	3.69	6.96	0.68	1.26
11	4.13	6.14	0.96	1.62
12	7.58	8.9	0.63	1.2
13	2.26	1.24	0.79	1.55
14	3.68	2.6	0.64	1.56
15	3.87	2.98	0.65	1.32
16	3.32	1.37	0.68	0.91

Median (*n* = 13)	3.65	2.60	0.68	1.32
*P value (pre- versus posttreatment)	0.30		<0.0001	

*Mann-Whitney *U* test, 2-tailed; pretreatment versus posttreatment values.
